# Precise Structure and Anticoagulant Activity of Fucosylated Glycosaminoglycan from *Apostichopus japonicus*: Analysis of Its Depolymerized Fragments

**DOI:** 10.3390/md17040195

**Published:** 2019-03-27

**Authors:** Ruowei Guan, Yuan Peng, Lutan Zhou, Wenqi Zheng, Xixi Liu, Pin Wang, Qingxia Yuan, Na Gao, Longyan Zhao, Jinhua Zhao

**Affiliations:** 1 School of Pharmaceutical Sciences, South-Central University for Nationalities, Wuhan 430074, China; ruoweiguan13@163.com (R.G.); m17671716278@163.com (Y.P.); zwq_scuec@126.com (W.Z.); 15927391554@163.com (X.L.); pinwang1994@163.com (P.W.); qingxiayuan@mail.scuec.edu.cn (Q.Y.); 2 State Key Laboratory of Phytochemistry and Plant Resources in West China, Kunming Institute of Botany, Chinese Academy of Sciences, Kunming 650201, China; zhoulutan@mail.kib.ac.cn; 3 University of Chinese Academy of Sciences, Beijing 100049, China

**Keywords:** *Apostichopus japonicus*, fucosylated glycosaminoglycan, oligosaccharide, unambiguous structure, anti-Fxase

## Abstract

*Apostichopus japonicus* is one of the most economically important species in sea cucumber aquaculture in China. Fucosylated glycosaminoglycan from *A. japonicus* (AjFG) has shown multiple pharmacological activities. However, results from studies on the structure of AjFG are still controversial. In this study, the deaminative depolymerization method that is glycosidic bond-selective was used to prepare the depolymerized products from AjFG (dAjFG), and then a series of purified oligosaccharide fragments such as tri-, hexa-, nona-, and dodecasaccharides were obtained from dAjFG by gel permeation chromatography. The 1D/2D NMR and ESI-MS spectrometry analyses showed that these oligosaccharides had the structural formula of l-FucS-α1,3-d-GlcA-β1,3-{d-GalNAc_4S6S_-β1,4-[l-FucS-α1,3-]d-GlcA-β1,3-}*_n_*-d-anTal-diol_4S6S_ (*n* = 0, 1, 2, 3; FucS represents Fuc_2S4S_, Fuc_3S4S_, or Fuc_4S_). Thus, the unambiguous structure of native AjFG can be rationally deduced: it had the backbone of {-4-d-GlcA-β1,3-d-GalNAc_4S6S_-β1-}*_n_*, which is similar to chondroitin sulfate E, and each d-GlcA residue in the backbone was branched with a l-FucS monosaccharide at *O*-3. Bioactivity assays confirmed that dAjFG and nonasaccharides and dodecasaccharides from AjFG had potent anticoagulant activity by intrinsic FXase inhibition while avoiding side effects such as FXII activation and platelet aggregation.

## 1. Introduction

Sea cucumber *Apostichopus japonicus* (or *Stichopus japonicus*) is widely distributed in the northwest Pacific, e.g., northern coast of China and almost all coastal areas of Japan, and has become one of the most popular sea cucumber species around the world due to its high value as a marine tonic [1]. Studies on this marine animal showed that it contains valuable nutrients, such as collagens, peptides, amino acids, sphingolipids, and polysaccharides [2]. Fucosylated glycosaminoglycan (FG) and fucan sulfate (FS) are the two main polysaccharides found in the body wall of sea cucumber. FG is a structurally distinctive glycosaminoglycan derivative containing fucose sulfate (FucS) branches found up to now exclusively in sea cucumbers, and has attracted great attention for its broad bioactivities such as anticoagulant, antithrombotic, anticancer, anti-human immunodeficiency-virus, anti-inflammatory, and immunomodulatory activities [3,4,5].

In the past four decades, a series of investigations on the structure of FG from *A. japonicus* (AjFG) have been carried out, but the conclusions are inconsistent. In 1980, that AjFG consisted of three types of monosaccharide—glucuronic acid (GlcA), *N*-acetylgalactosamine (GalNAc), and fucose (Fuc)—was reported for the first time [6]. In 1990, a study from Japan showed that AjFG had a chondroitin sulfate (CS)-like backbone (CS, CS-A, CS-C, and CS-E), and the trisaccharide (fucotriose) side chains linked to the backbone [7]. A later research showed that AjFG had three types of FucS residues, Fuc 2,4-disulfate (Fuc_2S4S_), 3,4-disulfate (Fuc_3S4S_), and 4-monosulfate (Fuc_4S_) with a molar ratio of 5:3:1, which linked to C-3 of GlcA residues in the CS-like backbone, and GalNAc residues in the backbone were sulfated at both *O*-4 and *O*-6 positions [8]. Based on methylation analysis, it was suggested that the FucS branches were present as the disaccharides (fucobioses) which linked to the backbone via (1→3) linkages, and the branches stretched not only from *O*-3 of GlcA but also from *O*-4 or *O*-6, or both, of GalNAc in the backbone [9]. In recent years, studies on the structure of AjFG have made great advances, but controversy remains on the glycosidic linkage sites of FucS branches and the sulfation patterns of GalNAc and Fuc. In 2015, another research also proposed that the FucS branches linked to both *O*-3 of GlcA and *O*-4/6 of GalNAc in the backbone [10]. In 2016, however, the study by Ustyuzhanina et al. showed that the backbone of AjFG was CS-A- or CS-E-linked at a molar ratio of about 2:1, and the Fuc_2S4S_, Fuc_3S4S_, and Fuc_4S_ residues only linked to *O*-3 of GlcA in the backbone [11]. Meanwhile another researcher suggested that the GalNAc residues in the backbone were all 4,6-disulfated and the branches only consisted of Fuc_2S4S_ and Fuc_3S4S_ residues [12]. Above all, the exact structure of AjFG still needs to be clarified.

AjFG displays a wide spectrum of pharmacological activities, including anticoagulant, antithrombotic, anti-inflammatory, neuroprotective, antihyperlipidemic activities [12,13,14,15]. Moreover, the activities are associated with its structural features such as sulfation patterns of Fuc branches and molecular mass [12,16]. Apparently, the exact structural analysis of AjFG is fundamental for investigating pharmacological mechanism and structure–activity relationship, which favors the exploration of its application value.

The lack of consensus on the structure of AjFG is mainly due to the limitations of the present techniques and strategies. In order to obtain FG fragments for structural analysis, partial acid hydrolysis was used to degrade FG, but it resulted in the loss of FucS sidechains and sulfate groups [17,18]. Hydrogen peroxide depolymerization was another method for FG degradation, but its exact cleavage position in polysaccharide chain remains unclear to date. Additionally, the high sulfation of AjFG hinders its complete methylation, and further its structural analysis [18].

In recent years, the glycosidic bond-selective methods, such as deacetylation–deaminative cleavage and β-eliminative depolymerization, have been established by our group, and applied for elucidating structures of some FGs that contained only one type of FucS branches [16,19,20]. In this study, the deacetylation–deaminative cleavage, which could selectively cleave the glycosidic bond at the GalNAc position, was used to prepare the depolymerized products from AjFG (dAjFG). The oligosaccharides with various degree of polymerization (dp) separated from dAjFG were then analyzed by 1D/2D NMR (^1^H, ^13^C, COSY, TOCSY, ROESY, HSQC, and HMBC) and electrospray ionization quadrupole time-of-flight mass spectrometry (ESI-Q-Tof-MS) spectrometry. Based on this “bottom-up” strategy, the precise structure of AjFG can be determined reliably. Moreover, bioactivity assays showed that AjFG and dAjFG had potent anticoagulant and anti-FXase activities, while they could also activate factor XII and induce platelet aggregation [4,15]. Therefore, in this study, we further investigated the structure–activity relationship by analysis of a series of oligosaccharides with various dp.

## 2. Results and Discussion

### 2.1. Extraction, Isolation, and Purification of AjFG

**Figure 1:**
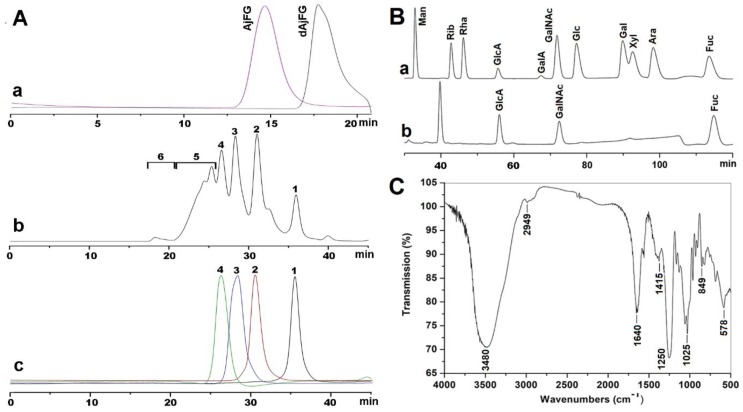
Physicochemical properties of AjFG, dAjFG, and the purified fragments. HPLC profiles of AjFG (**a**), dAjFG (**a** and **b**) and the oligosaccharide fragments with various dp (**c**) (**A**); chromatograms of PMP derivatives of mixed monosaccharide standards (**a**) and AjFG (**b**) (**B**); and FI-IR spectrum of AjFG (**C**).

Enzymatic digestion combined with alkali treatment used in this study is a common method for extracting FG, which is consistent with the extraction method used for isolation of AjFG by other groups [6,7,21]. After purification, the purity of AjFG was >99%, as determined by high-performance gel permeation chromatography (HPGPC) using a Shodex OH-pak SB-804 HQ column. The HPGPC profile of AjFG showed a single homogeneous peak (Figure 1A-a). The yield of AjFG was about 0.3%, which is slightly higher than that (about 0.2%) in a previous report [11]. No ultraviolet absorptions around 260 and 280 nm were detected by a UV-detector, indicating absence of nucleic acids, peptides, and proteins in AjFG. ^1^H-NMR spectrum (Figure S1A) of AjFG is similar to those reported previously [8,10,11,12].

### 2.2. Analysis of Physicochemical Properties

According to the calibration curve, the weight-average molecular weight (M_w_) of AjFG was estimated to be 76.4 kDa. Monosaccharide composition analysis showed that AjFG was composed of three types of monosaccharides—GlcA, GalNAc, and Fuc—with the molar ratio of 1:0.94:1.07 (Figure 1B), which was consistent with the reported data [6]. The specific rotation of AjFG was determined to be −68°. The molar ratio of sulfate to uronic acid groups in AjFG was 3.59, as calculated from its conductometric titration (Figure S2). These negatively charged groups in the AjFG chain may contribute to its pharmacological activities [22].

The FT-IR spectrum of AjFG was shown in Figure 1C. Broad and strong absorption bands at around 3480 cm^−1^ could be assigned to the stretching vibrations of the OH group. Absorption at 2949 cm^−1^ for the C-H stretching vibrations of CH_3_ was also observed. Absorption at 1640 cm^−1^ was due to the asymmetric stretching vibration of C=O of d-GlcA and d-GalNAc. Absorption at 1415 cm^−1^ was derived from the symmetric stretching vibration of COO^−^ of d-GlcA. Absorption at 1025 cm^−1^ was ascribed for the stretching vibrations of C-O. In addition, the absorption peaks at 1250 and 849 cm^−1^ were assigned to the stretching vibration of S=O of the sulfate group and the bending vibration of C-O-S of the sulfate group in an axial position, respectively [16]. These signals observed in FT-IR spectrum were in agreement with the results of their chemical composition analyses.

The glycosidic linkage of AjFG was tentatively determined by methylation method as reported in the literature [9]; however, the absorption peaks at around 3100 cm^−1^ still existed in the FT-IR spectrum even after seven repeats of methylation. The result indicated that AjFG was difficult to be completely methylated, which may be due to the steric hindrance effects of FucS sidechains and high contents of sulfate esters in AjFG. These results suggested that the methylation method might not be suitable for structural analysis of FG for the incomplete methylation might lead to structural misjudgment.

### 2.3. Preparation of Oligosaccharides with Various dp

^1^H NMR spectra (Figure S1) showed overlapped signals for AjFG and dAjFG due to their large molecular size, which resulted in the difficulty in the structural analysis. Although enzymatic cleavage of glycosaminoglycans (GAGs) is an effective method to prepare oligosaccharides [23], unlike linear GAGs, FGs cannot be directly degraded by the known polysaccharide hydrolyase such as heparanase or chondroitin lyase [21,24]. Fortunately, our group has established the method of partial deacetylation–deaminative cleavage, which can selectively cleave the glycosidic linkage at the site of GalNAc and form 2,5-anhydro-d-talose-diol (anTal-diol) residues at the reducing ends of resulting fragments [16].

Besides FGs, other polysaccharides, such as FS and neutral glycan, are present in sea cucumber [25,26]. As shown in Figure 1A-b, the polysaccharides (fraction 6) that contained no hexosamine were not degraded by deacetylation–deaminative cleavage, and thus their interference in the structural determination of AjFG could be avoided. The method of deacetylation–deaminative cleavage was applied to depolymerize AjFG to obtain dAjFG. The M_w_ of dAjFG was determined as 3.90 kDa using the Shodex OH-pak SB-804 HQ column (Figure 1A-a). HPLC profiles of dAjFG, using Superdex Peptide 10/300 GL column, showed that dAjFG was composed of several fragments with different sizes (Figure 1A-b). Fragment 1–Fragment 4 (Fr-1–Fr-4) were further purified from dAjFG by repeated gel permeation chromatography (GPC) using Bio-Gel P10, P6 and P4 columns, which were identified to be tri-, hexa-, nona-, and dodecasaccharide, respectively (Figure 1A-c), according to the retention times of the standard purified oligosaccharides whose structures and M_w_ have been reported in our previous study [16].

### 2.4. NMR and ESI-Q-TOF-MS Analysis

In the ^1^H NMR spectrum of AjFG (Figure S1A), the signals observed at 1.30 ppm and 1.99 ppm could be assigned to methyl protons of FucS and GalNAc (-(CO)CH_3_) residues, respectively. The broad and overlapping signals at the region of 3.5–5.0 ppm could be assigned to the ring protons and anomeric protons of GalNAc and GlcA. The signals at 5.29, 5.34, and 5.63 ppm could be assigned to the anomeric protons of FucS residues, indicating that AjFG may contain three types of FucS residues. In the ^1^H NMR spectrum of dAjFG (Figure S1B), the signals of both anomeric and methyl proton of FucS were also overlapping, despite in less extent than those of AjFG. These overlapped signals of AjFG and dAjFG caused difficulty in their structural analyses. In contrast, the exact structures of oligosaccharides, isolated from the dAjFG, could be elucidated by 1D and 2D NMR spectra, and further confirmed by ESI-Q-TOF-MS analysis. 

**Figure 2:**
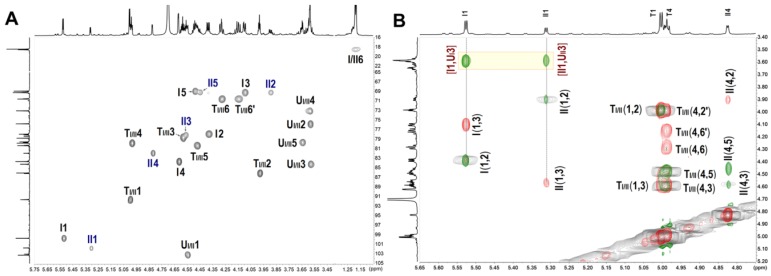
^1^H-^13^C HSQC (**A**) and superimposed ^1^H-^1^H COSY (black), TOCSY (red), and ROESY (green) (**B**) spectra of Fragment 1 (Fr-1). I and II represent type I and II of FucS, respectively. U_I_, U_II_, T_I_, and T_II_ represent the GlcA and anTal-diol residues substituted with type I and II of FucS, respectively.

The 1D and 2D NMR spectra (Figure 2 & Figure S3) of Fr-1 showed that it contained two types of trisaccharides. According to the HSQC spectrum (Figure 2A), the anomeric C/H signals of types I and II of FucS (I and II) were at 99.7/5.53 and 101.8/5.31 ppm, respectively. The anomeric C/H signals of GlcA (U_I_ and U_II_) linked to I and II were at 103.3/4.56 and 103.1/4.55 ppm, respectively, while the signals at 91.7/5.00 and 91.6/5.00 ppm could be assigned to anomeric C/H signals of anTal-diol (T_I_ and T_II_) linked to U_I_ and U_II_, respectively [16,20]. The complete assignments of the protons and carbons are given in Table 1. The superimposed COSY, TOCSY, ROESY spectra (Figure 2B) and HMBC spectrum (Figure S3D) clearly showed that I and II contained in Fr-1 linked to U_I_ and U_II_ by α1,3-glycosidic bonds, and no FucS linked to anTal-diol. According to the chemical shift values of the ring C/H signals of FucS and GalNAc, I and II were identified as Fuc_2S4S_ and Fuc_3S4S_, respectively, and both *O*-4 and *O*-6 positions of GalNAc were substituted by sulfate groups. The small H-H coupling constants (3.92 and 3.98 Hz) indicated the presence of an α-linkage between Fuc and GlcA. Additionally, ESI-Q-TOF-MS analysis showed the *m/z* values of 908.9023 and 886.9204 for [M − Na]^−^ (calculated as 908.9000) and [M − 2Na + H]^−^ (calculated as 886.9100), respectively, confirming that the molecular formula of Fr-1 was C_18_H_25_O_28_S_4_Na_5_ (Figure S8). Based on these analyses, the structure of Fr-1 was established as l-FucS-α1,3-d-GlcA-β1,3-d-anTal-diol_4S6S_ (FucS = Fuc_2S4S_ or Fuc_3S4S_), and the molar ratio of two types of trisaccharides is approximately 2:1 (Table 1).

Fr-2 is a mixture of hexoses, which showed more signals in the ^1^H and ^13^C NMR spectra (Figure S4A,D) than those of Fr-1. According to the HSQC (Figure S5A) spectrum, six sets of anomeric proton signals of FucS at about 5.20–5.65 ppm could be found in the ^1^H NMR spectrum. According to the superimposed COSY/TOCSY/ROESY spectra, Fr-2 contained three types of FucS (I, II and III) (Figure S5B). The ring C/H signals of FucS could be assigned according to the 2D NMR spectra (Table S1). The signals from the unreduced terminal GlcA (U) and the reduced terminal anTal-diol (Figure S5, Table S1) were similar to those from Fr-1 in the 1D and 2D NMR spectra. According to 2D NMR spectra, Fr-2 also contained a GlcA (U′) which linked to anTal-diol, in addition to the unreduced terminal GlcA. U and U′ were all connected by GalNAc. It could be confirmed by the ROESY and HMBC spectra that all FucS were α1,3-linked to U or U′ but not GalNAc (or anTal-ol) in Fr-2, and the branches were FucS monosaccharides but not fucodioses or fucotrioses. Based on these analyses, the structure of Fr-2 was established as l-FucS-α1,3-d-GlcA-β1,3-d-GalNAc_4S6S_-β1,4-[l-FucS-α1,3-]-d-GlcA-β1,3-d-anTal-diol_4S6S_ (FucS = Fuc_2S4S_ ~51%, Fuc_3S4S_ ~30% and Fuc_4S_ ~19%). The structure was further confirmed by the ESI-Q-TOF-MS spectrum (Figure S8). The ESI-Q-TOF-MS of Fr-2 afforded the *m/z* values of 898.4313 and 847.9929 for [M_1_ − 4Na + 2H]^2−^ (calculated as 898.4266, C_38_H_51_O_5__5_N_1_S_8_Na_10_) and for [M_2_ − 4Na + 2H]^2−^ (calculated as 847.4500, C_38_H_5__2_O_5__2_N_1_S_7_Na_9_), respectively.

Similarly, according to the 1D/2D NMR and ESI-Q-TOF-MS spectra of Fr-3 and Fr-4 (Figures S4, S6 and S8), their structures were deduced to be l-FucS-α1,3-d-GlcA-β1,3-{d-GalNAc_4S6S_-β1,4-[l-FucS-α1,3]d-GlcA-β1,3-}*_n_*-d-anTal-diol_4S6S_ (*n* = 2, 3). They both contained three types of FucS (Figure S4 and Table S2).

Fr-1–Fr-4 had a common structural formula of l-FucS-α1,3-d-GlcA-β1,3-{d-GalNAc_4S6S_-β1,4-[l-FucS-α1,3-]d-GlcA-β1,3-}*_n_*-d-anTal-diol_4S6S_ (*n* = 0–3; FucS = Fuc_2S4S_, Fuc_3S4S_ or Fuc_4S_). In these fragments, the reduced terminal anTal-diol was produced by deacetylation–deaminative cleavage of GalNAc at the reducing end. As shown in Figure 1A-b, Fr-1–Fr-4 with the yield of about 60% was the main component of dAjFG, which contained almost all oligosaccharide fragments with M_w_ less than 3.49 kDa in dAjFG. The high-molecular-weight fraction 5 accounted for about 29% in dAjFG showed all chemical signals present in Fr-1–Fr-4, but did not present additional unknown H and C signals (Figure S7). Therefore, based on the “bottom-up” strategy, the structure of native AjFG could be rationally deduced as a repeating trisaccharide unit -{4-[l-FucS-α1,3]-d-GlcA-β1,3-d-GalNAc_4S6S_-β1}-, which possessed a CS-E-like backbone and a monosaccharide FucS sidechain that was connected to GlcA of the backbone by the α1,3-glycosidic bond.

Over the past 40 years, several groups have conducted a systematic research on the structure of AjFG. However, their conclusions are not consistent and some controversies remain to be solved, including whether di- or trisaccharide sidechains are in AjFG, whether some FucS sidechains link to GalNAc, and whether C-4 or C-6 position of GalNAc is not sulfated [7,8,9,10,11,12]. Results of this study clearly showed that all FucS side chains in AjFG existed as a monosaccharide type, and the fucobiose or fucotriose sidechains were not present. The FucS sidechain only attached to GlcA by the α1,3-glycosidic bond, and no FucS sidechain linked to GalNAc. Furthermore, *O*-4 and *O*-6 positions of the GalNAc were all substituted by sulfate groups, and monosulfated GalNAc was not observed.

The results of this study showed that although several sulfated types of FucS existed in the native AjFG, the oligosaccharides produced by the deacetylation–deaminative cleavage still had regular structure. The results indicated that the depolymerization had no significant effects on the chemical structures of AjFG except that the partial deacetylation–deaminative cleavage occurred at the site of GalNAc.

Fr-2–Fr-4 all contained three types of FucS, and the ratio was similar to the native AjFG. For example, the molar ratio of I, II, and III was about 52:29:19 in Fr-2, which was similar to that of 54:29:17 in the AjFG. Fr-1 did not contain the sidechain of Fuc_4S_, which might be due to the low content of Fuc_4S_ in the native AjFG. For the trisaccharides, the decrease in M_w_ due to less sulfate groups might make it easier to be separated by gel chromatography.

### 2.5. Analysis of the Anticoagulant Activity

Among the biological activities of AjFG, the anticoagulant activity and intrinsic FXase inhibition activity have attracted most attention [10]. However, the structure-anti-FXase activity relationship of AjFG and its derivatives needs to be investigated. In our previous studies, various FG oligosaccharides with a single type of FucS from FG, obtained from other sea cucumber species, showed potent anticoagulant activity through selectively inhibiting intrinsic FXase. Unlike native FG, these FG oligosaccharides caused no significant FXII activation and platelet aggregation [16,19,20].

**Figure 3:**
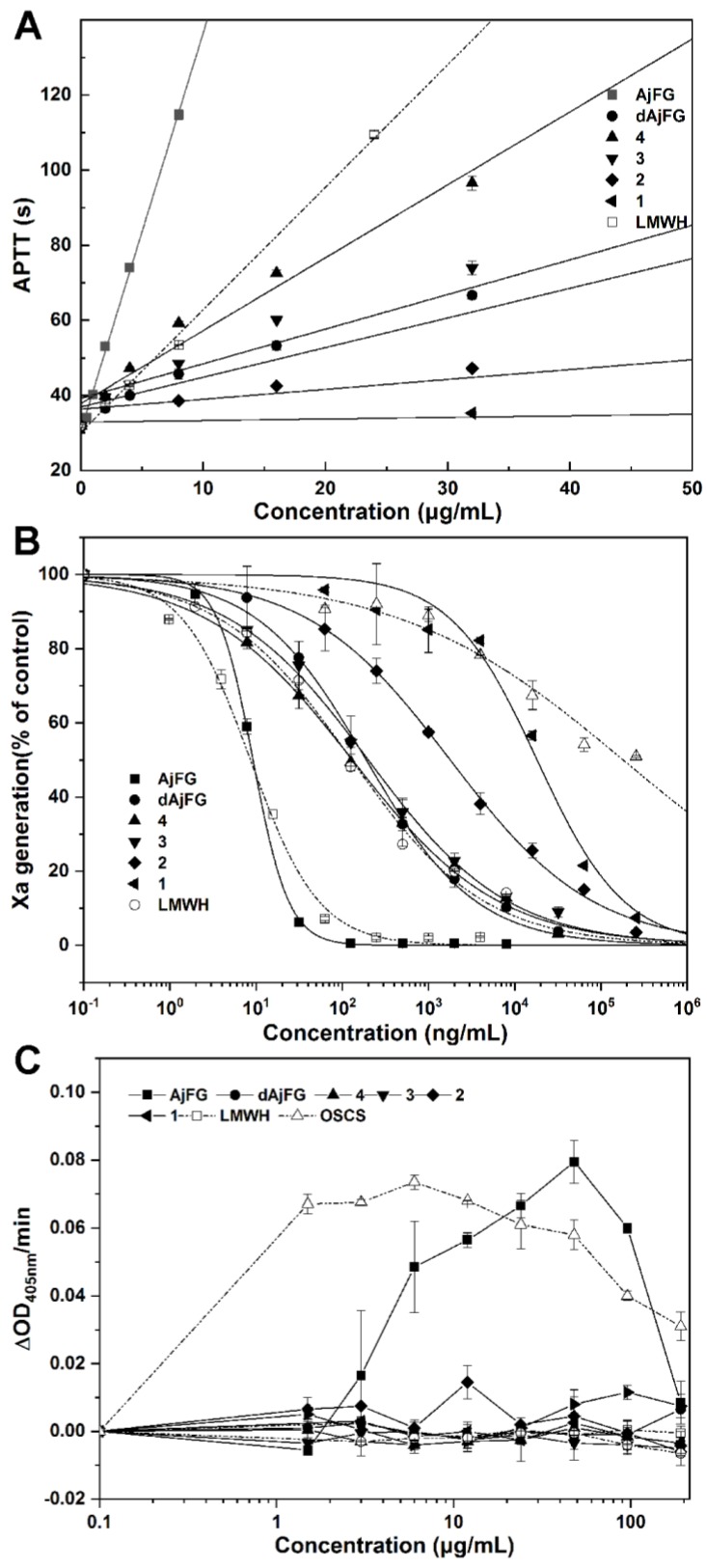
The effects of AjFG, dAjFG and oligosaccharides with various dp (Fr-1–Fr-4) on APTT (**A**), intrinsic FXase (**B**), and human factor XII activation (**C**). The results were expressed as mean ± SD (*n* = 2).

In this study, the anticoagulant activities of oligosaccharides from AjFG were assessed by the activated partial thromboplastin time (APTT) and intrinsic FXase inhibition assays, compared with low-molecular-weight heparin (LMWH) (Figure 3A,B, Table 2). The native AjFG showed more potent APTT prolonging activity than LMWH, and its concentration (3.06 μg/mL) required for double APTT was about 1/3 of that of LMWH (11.3 μg/mL). The concentrations of dAjFG, Fr-3, and Fr-4 required for double APTT were 26.5, 10.3 and 20.1 μg/mL, respectively, which exhibited comparable or less potent APTT prolonging activity than LMWH. However, Fr-1 and Fr-2 showed no significant activity at the concentration up to 128 μg/mL. These results indicated that AjFG, dAjFG, Fr-3, and Fr-4 had strong inhibitory effects on intrinsic coagulation pathway, which was chain length-dependent.

Additionally, the structure–activity relationship of AjFG and its derivatives for intrinsic FXase inhibition was in a similar manner to their APTT prolonging activity (Figure 3A,B, Table 2). The anti-FXase activity of AjFG was more than 10-fold higher than that of LMWH, while the activities of dAjFG, Fr-3 and Fr-4 were slightly weaker than that of LMWH. However, Fr-1 and Fr-2 had no significant anti-FXase activity at concentrations as high as 1000 μg/mL. These results indicated that three trisaccharide structural units might be the minimum structural fragment of AjFG for its potent anti-FXase and anticoagulant activities.

The application of FGs as a novel antithrombotic is severely limited due to their undesirable effects, such as factor XII and platelet activation [27,28]. Native AjFG with large M_w_ exhibited FXII activation activity, which might cause procoagulant effect or inflammatory reactions [29,30]. The FXII activation activities of AjFG and its derivatives were evaluated, and the over-sulfated chondroitin (OSCS) was used as the positive control. Compared with OSCS and AjFG, Fr-1–Fr-4 exhibited no FXII activation within the experimental concentration range (Figure 3C). Previous studies have shown that the native AjFG induces clumping of human, rat, rabbit, and mouse platelets, and causes a dramatic decrease in the platelet count [31,32]. In our present study, compared with ADP, Fr-1–Fr-4 did not exhibit platelet aggregation (*P* > 0.05) (Figure S9).

In previous study, a fragment of AjFG named DHG was produced by hydrogen peroxide depolymerization, it had the M_w_ of approximately 8–15 kD and dp of about 25–50, and its anticoagulant activity was investigated [33,34]. In this study, the purified oligosaccharides with a dp of 9 (M_w_ 2.52–2.83) displayed strong anticoagulant and anti-FXase activity. Comparison of the activities of AjFG, dAjFG, and the oligosaccharides with various dp enabled us to study their structure-activity relationship.

## 3. Materials and Methods

### 3.1. Materials and Chemicals

Dried sea cucumber *A. japonicus* was purchased from Dalian, Liaoning Province, China. Amberlite FPA98Cl was from Rohm and Haas Company (USA). Sepharose CL-6B and Superdex Peptide 10/300 GL columns (10 mm × 300 mm) were from GE Healthcare Life Sciences (Uppsala, Sweden). Bio-Gel P-2, P4, P-6, and P-10 columns were from Bio-Rad Laboratories (USA). Papain (800 U/mg) was from Shanghai Yuanye Bio-Technology Co., Ltd. (Shanghai, China). Standard SEC-Pullulan was from Sepax Technologies Inc. (Delaware, America). l-Rhamnose (Rha), d-galactose (Gal), d-galacturonic acid (GalA), d-glucose (Glc), GlcA, 3-methyl-1-phenyl-2-pyrazolin-5-one (PMP) were from Sigma Chemical Co. (St. Louis, MO, USA). d-Mannose (Man), l-arabinose (Ara), d-ribose (Rib), d-xylose (Xyl), GalNAc, and l-Fuc were from Aladdin Chemical Reagent Co., Ltd. (Shanghai, China). LMWH (Enoxaparin, 0.4 mL × 4000 AXaIU) was from Sanofi-Aventis (France). OSCS was from Serva Electrophoresis GmbH (Germany). ADP, kallikrein chromogenic substrate CS-31 (02) and FIXa chromogenic substrate CS-51 (09) were from Hyphen Biomed (France). The APTT, CaCl_2_, and coagulation control plasma were from TICO GmbH (Germany). Human factor VIII was from Bayer Healthcare LLC (Germany) Biophen FVIII: C kit was from Hyphen Biomed (France). Human factor XII was from Assaypro (USA). All other chemicals used were of analytical grade.

### 3.2. Extraction, Isolation and Purification of AjFG

AjFG was extracted by a method as previously described with minor modifications [7]. The dried and minced body walls of *A. japonicus* (1300 g) were treated with 0.1% papain aqueous solution at 50 °C for 6 h and with 0.5 M NaOH at 60 °C for 2 h. The proteins in extracting solution were removed by adding 6 M HCl to adjust a pH to 2.8, standing overnight at 4 °C and centrifuging at 4700 rpm for 15 min. The crude polysaccharides were further purified by ion-exchange chromatography with an Amberlite FPA98 ion exchange resin (8 cm × 50 cm) followed by gel permeation chromatography with a Sepharose CL-6B (2.0 cm × 150 cm). Finally, the AjFG fractions were combined, dialyzed, and lyophilized. The yield of purified AjFG was about 4.2 g.

### 3.3. Determination of Physicochemical Properties

The molecular weights of AjFG and dAjFG were determined by HPGPC using an Agilent 1260 series apparatus (Agilent Technologies, Santa Clara, CA, USA) equipped with a Shodex OH-pak SB-804 HQ column and a RID detector. Chromatographic procedures were performed according to previous method [25]. A standard curve of molecular weight was calculated with the SEC-Pullulan standards and calibrated with the dodecasaccharide obtained from the sea cucumber *S. variegatus* with known relative M_w_ [16]. The monosaccharide compositions were measured as described previously [35], and the PMP-labeled saccharides were analyzed by an Agilent 1260 HPLC system equipped with an Eclipse Plus C18 column (4.6 × 250 mm, 5 μm, Agilent) and a DAD detector. The -OSO^3−^ to -COO^−^ molar ratio of AjFG was determined by a conductimetric titration method [36]. The specific rotation of AjFG was detected by a polarimeter at the concentration of 0.5 mg/mL at 20 °C. The IR spectrum of AjFG was measured through KBr pellet by Bruker Tensor 27 infrared spectrometer (Ettlingen, Germany) at the range of 4000–400 cm^−1^. Methylation of AjFG was carried out according to the literature [9,10].

### 3.4. Preparation and Isolation of Oligosaccharides of AjFG

AjFG was depolymerized by the partial deacetylation–deaminative cleavage method as described previously [16]. Briefly, AjFG (2000 mg) was deacetylated by hydrazine monohydrate containing 1% hydrazine sulfate at 90 °C for 24 h. Then, the partial deacetylated sample (1806 mg) was cleaved with nitrous acid at room temperature for 10 min, dialyzed with a molecular weight cut-off of 100–500 Da (Spectrum Laboratories Inc., Ft. Lauderdale, FL, USA) and lyophilized. The yield of dAjFG was 1427 mg. The dAjFG (1000 mg) were separated by repeated GPC using bio-gel P10, P6, and P4 columns and eluted with 0.2 M NaCl to obtain a range of size-homogeneous oligosaccharides. The size homogeneity could be determined by analytical HPLC equipped with a Superdex Peptide 10/300 GL column. Fr-1–Fr-4 were desalted on a column which packed with Sephadex G-10 and lyophilized [26]. The yields of Fr-1–Fr-4 were 69, 180, 186, and 164 mg, respectively, and the yield of the fraction 5 with high molecular weight was 295 mg.

### 3.5. NMR and ESI-Q-TOF-MS Analysis

The purified polysaccharide and oligosaccharide fragments were dissolved in deuterium oxide (D_2_O, 99.9% D) and lyophilized for three times to replace exchangeable protons with D_2_O. The lyophilized samples were then dissolved in D_2_O at a concentration of 40 mg/mL for NMR analyses. The NMR analyses were performed at 298 K with a Bruker Avance spectrometer of 800 MHz equipped with a ^13^C/^1^H dual probe in FT mode, and ^1^H-^1^H COSY, TOCSY, ROESY, ^1^H-^13^C HMBC, and HSQC spectra were recorded using state–time proportional phase incrementation for quadrature detection in the indirect dimension. Negative-ion ESI-MS was performed on a Thermo Q Exactive mass spectrometer. The MS spectrometric conditions were as follows: ESI in negative ion mode, spray voltage of 3800 V, sheath gas flow rate of 40.0 L/min, and aux gas flow rate of 20.0 L/min. The mass spectra of the oligosaccharides were acquired in scan mode (*m/z* scan range 600–4000). Data were analyzed using Thermo Xcalibur 4.0.27.19 software.

### 3.6. Determination of Anticoagulant Activities In Vitro

APTT were determined with a coagulometer (TECO MC-4000, Germany) using APTT reagents and coagulation control plasma as previously described [37]. Inhibition of intrinsic factor Xase complex was determined as previously described [38]. Different concentrations of samples (AjFG, dAjFG, Fr-1–Fr-4) were incubated with 2 IU/mL factorc VIII (30 μL), and 60 nM factor IXa (30 μL) (containing human thrombin, calcium, and synthetic phospholipids) in a total volume of 90 μL, for 2 min at 37 °C. The reaction was initiated by the addition of 50 nM factor X (30 μL) for 1 min at 37 °C. The amount of factor Xa formed was determined after 30 μL of 8.40 mM factor Xa chromogenic substrate SXa-11 was added to the reaction mixture, based on the absorbance at 405 nm. The OD_405_ nm was recorded at 37 °C using a Bio-Tek Microplate Reader (ELx808, BioTek^®^ Instruments, Inc., Winooski, VT, USA).

### 3.7. Human Factor XII Activation and Platelet Aggregation Assays

The activation of human factor XII in the presence of samples (AjFG, dAjFG, Fr-1–Fr-4) was determined as previously described [28]. Turbid metric measurements of platelet aggregation of polysaccharides were performed in a Chronolog Model 700 Aggregometer (Chronolog Corporation, Havertown, PA, USA), according to previous method [39]. After preparation of platelet-rich plasma (PRP) and platelet-poor plasma (PPP), platelet counts were adjusted by adding PPP to PRP to achieve a count of 250 × 10^9^ L^−1^. Then, PRP and PPP were positioned in testing places. Changes in optical density for platelet aggregation were recorded.

## 4. Conclusions

In this study, tri-, hexa-, nona-, and dodecasaccharides from AjFG were prepared by the partial deacetylation–deaminative cleavage method combined with the gel chromatography, and their structures were established by NMR and ESI-Q-TOF-MS spectrometry. Structural analysis of the oligosaccharide with various dp revealed the precise structure of a native AjFG as a polysaccharide composing of regular repeating trisaccharide units. AjFG comprises a backbone of CS-E, and branched with various monosaccharide FucS including Fuc_2S4S_ (~54%), Fuc_3S4S_ (~29%), and Fuc_4S_ (~17%), which linked to the same position, i.e., GlcA of the backbone via an α1,3-linkage. The *O*-4 and *O*-6 positions of GalNAc are all substituted by sulfate groups. Di- or trisaccharide Fuc branches and any Fuc sidechain linked to GalNAc were not found in AjFG. Anticoagulant assay confirmed that AjFG, dAjFG, nonasaccharides, and dodecasaccharides all possessed strong anticoagulant activity and intrinsic FXase inhibition might be the main mechanism. Particularly, nonasaccharides and dodecasaccharides exhibited negligible FXII activation and platelet aggregation activities, and might be the ideal anticoagulants. These data can provide a significant reference for the structure–activity relationship investigation and the development and application of AjFG.

## Figures and Tables

**Table 1:** ^1^H/^13^C NMR chemical shift assignments of Fr-1 (800 MHz, D_2_O).

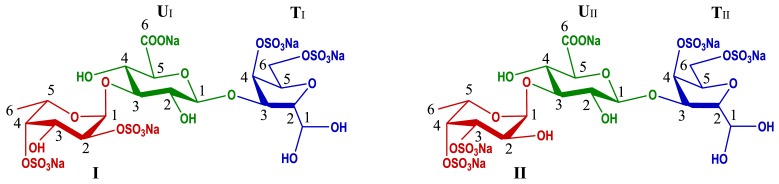											
	δH (ppm)		Coupling Constant in Hz	δC (ppm)			δH (ppm)		Coupling Constant in Hz	δC (ppm)	
T_I_	H-1	5.00	*J*_(1,2)_ = 4.41	C-1	91.7	T_II_	H-1	5.00	*J*_(1,2)_ = 4.41	C-1	91.6
	H-2	3.98	*J*_(2,3)_ = 4.52	C-2	86.1		H-2	3.99	*J*_(2,3)_ = 4.52	C-2	86.2
	H-3	4.59	*J*_(3,4)_ = 5.20	C-3	78.8		H-3	4.60	*J*_(3,4)_ = 5.06	C-3	78.4
	H-4	**4.9** **9**	*J*_(4,5)_ = 5.10	C-4	**79.8**		H-4	**4.99**	*J*_(2,3)_ = 5.13	C-4	**79.7**
	H-5	4.48	*J*_(5,6′)_ = 9.10	C-5	80.4		H-5	4.48	*J*_(5,6′)_ = 9.10	C-5	80.3
	H-6	**4.2** **9**	*J*_(5,6)_ = 2.50	C-6	**70.** **6**		H-6	**4.27**	*J*_(5,6)_ = 2.47	C-6	**70.5**
	H-6′	**4.1** **5**	*J*_(6,6′)_ =11.29				H-6′	**4.16**	*J*_(6,6′)_ = 11.26		
**U_I_**	H-1	**4.56**	*J*_(1,2)_ = 7.74	C-1	**103.** **3**	**U**_II_	H-1	**4.55**	*J*_(1,2)_ = 7.44	C-1	**103.** **1**
	H-2	3.59	*J*_(2,3)_ = 8.29	C-2	75.8		H-2	3.59	*J*_(2,3)_ = 8.37	C-2	75.8
	H-3	**3.** **60**	*J*_(3,4)_ = 7.55	C-3	**84.2**		H-3	**3.5** **7**	*J*_(3,4)_ = 7.78	C-3	**84.** **3**
	H-4	3.60	*J*_(4,5)_ = 9.73	C-4	73.1		H-4	3.62	*J*_(4,5)_ = 9.38	C-4	73.1
	H-5	3.65		C-5	79.6		H-5	3.65		C-5	79.7
				C-6	178.4					C-6	178.4
**I**	H-1	**5.5** **3**	*J*_(1,2)_ = 3.92	C-1	**99.7**	**II**	H-1	**5.31**	*J*_(1,2)_ = 3.98	C-1	**101.** **8**
	H-2	**4.3** **9**	*J*_(2,3)_ = 10.58	C-2	**77.9**		H-2	3.90	*J*_(2,3)_ = 10.46	C-2	69.2
	H-3	4.10	*J*_(3,4)_ = 2.96	C-3	69.2		H-3	**4.5** **7**	*J*_(3,4)_ = 2.95	C-3	**78.1**
	H-4	**4.62**		C-4	**83.** **7**		H-4	**4.8** **3**		C-4	**81.9**
	H-5	4.50	*J*_(5,6)_ = 6.80	C-5	68.9		H-5	4.46	*J*_(5,6)_ = 6.62	C-5	69.2
	H-6	1.19		C-6	18.4		H-6	1.19		C-6	18.6

I, II, T_I_, T_II_, U_I_, U_II_ are as shown in the structural formula. Values in bold type indicate glycosylated or sulfated positions.

**Table 2:** Anticoagulant activity of AjFG, dAjFG, and Fr-1–Fr-4.

Sample	M_w_ (kDa)	APTT (μg/mL) ^a^	Anti-FXase (IC_50_, ng/mL) ^b^
AjFG	76.4	3.06	9.20
dAjFG	3.90	26.5	189
4	3.49	10.3	131
3	2.63	20.1	200
2	1.77	>128	>1000
1	0.93	>128	>1000
LMWH	4.50	11.3	128

^a^ The concentration required to double the APTT of human plasma; ^b^ IC_50_ values, the concentration required to inhibit 50% of protease activity.

## Supplementary Materials

The following are available online at https://www.mdpi.com/1660-3397/17/4/195/s1, 1D/2D NMR (^1^H, ^13^C, COSY, TOCSY, ROESY, HSQC, and HMBC) spectra, ESI-Q-Tof-MS spectra, ^1^H/^13^C NMR chemical shift assignments, and results of platelet aggregation assays of AjFG, dAjFG, and oligosaccharide fragments.
