# Supplementary material for: Precise Structure and Anticoagulant Activity of Fucosylated Glycosaminoglycan from Apostichopus japonicus: Analysis of Its Depolymerized Fragments
Source: Mar Drugs. 2019 Mar 27;17(4):195. doi: 10.3390/md17040195 (PMC6520811; doi:10.3390/md17040195)
Supplement: Supplementary file 1 [file marinedrugs-17-00195-s001.zip › marinedrugs-467526-SI.pdf]

Mar. Drugs **2019**, *17*, 195; doi:10.3390/md17040195

Supplementary data

Precise structure and anticoagulant activity of fucosylated glycosaminoglycan from *Apostichopus japonicus*: analysis of its depolymerized fragments

Ruowei Guan [1], Yuan Peng [1], Lutan Zhou [2, 3], Wenqi Zheng [1], Xixi Liu [1], Pin Wang [1], Qinxia Yuan [1], Na Gao [1, *], Longyan Zhao [1, *] and Jinhua Zhao [1, 2, *]

[1] *School of Pharmaceutical Sciences, South-Central University for Nationalities, Wuhan 430074, China*

[2] *State Key Laboratory of Phytochemistry and Plant Resources in West China, Kunming Institute of Botany, Chinese Academy of Sciences, Kunming 650201, China*

[3] *University of Chinese Academy of Sciences, Beijing 100049, China*

* Corresponding author.

E-mail addresses: zhaojinhua@mail.kib.ac.cn (J. Zhao); zhaolongyan@mail.scuec.edu.cn (L. Zhao); gn2008.happy@163.com (N. Gao).

SUPPLEMENTAL FIGURES AND TABLES

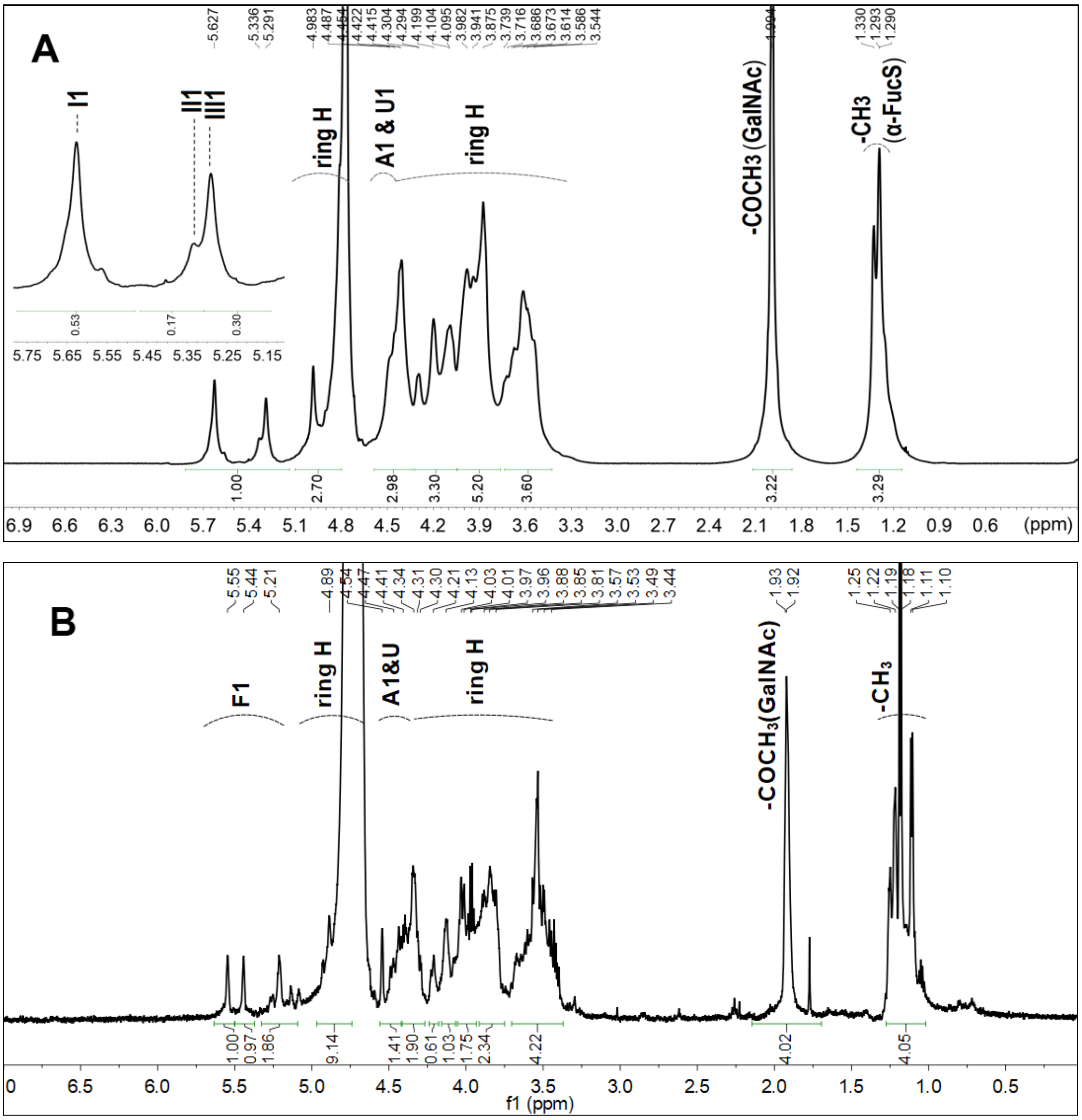

Figure S1. 1H-NMR spectra of AjFG (A) and dAjFG (B)

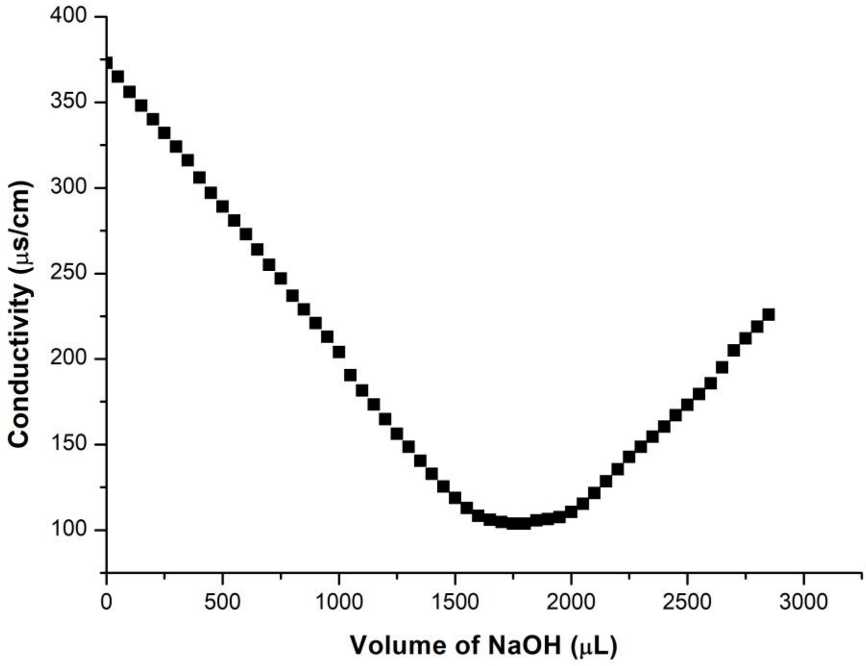

Figure S2. Conductometric titration curves of AjFG.

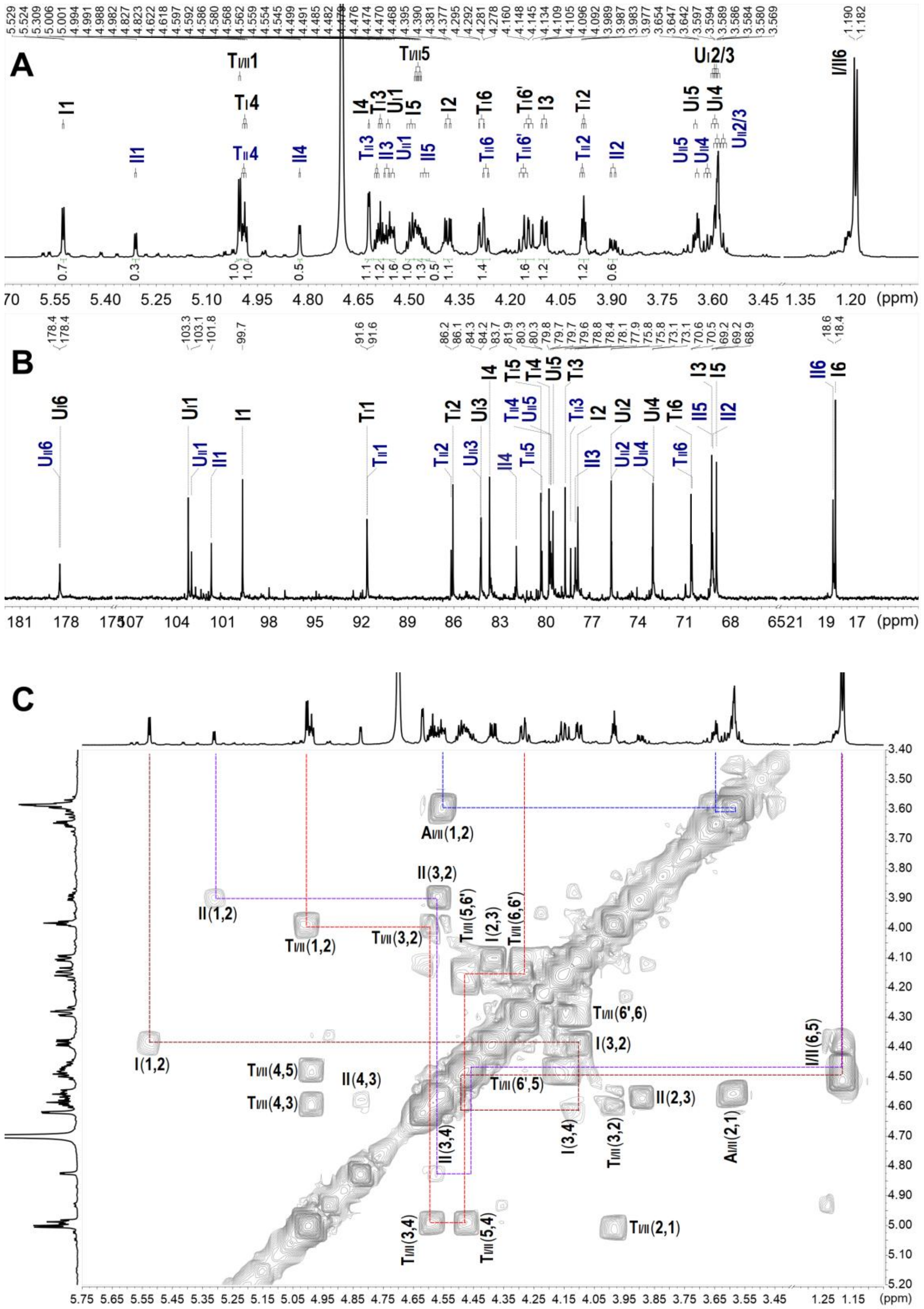
A
B
C
(ppm)
(ppm)
(ppm)
I1
II1
TI4
TII4
II4
I2
TI6
TII6
I3
TI2
TII2
II2
UI5
UI4
UII5
UII4
I/II6
UI6
UII6
UI1
UII1
II1
I1
TI1
TII1
TI2
TII2
UI3
UII3
I4
II4
T5
T4
UI5
T3
I2
UI2
UII2
UI4
UII4
TI6
TII6
I3
I5
II5
II2
II6
I6
II(3,2)
II(1,2)
I(1,2)
I(3,2)
II(4,3)
II(2,3)
I(3,4)
II(3,4)

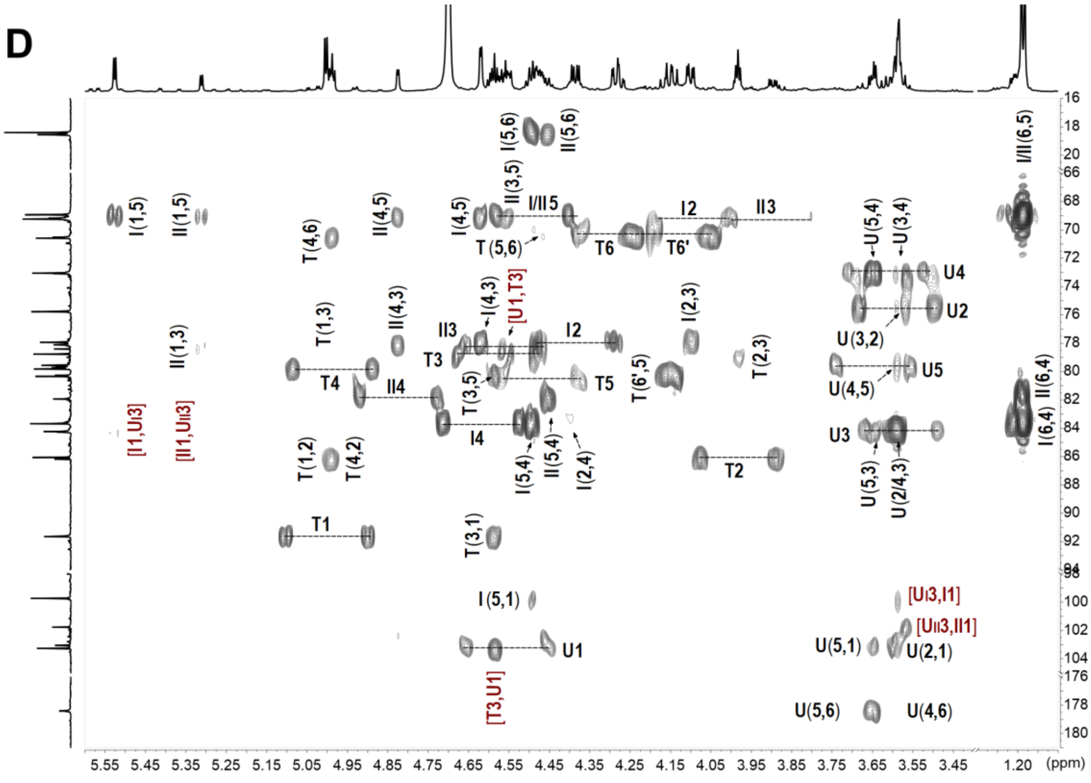

Figure S3. ^{1}H NMR (A),^{13}C NMR (B), ^{1}H-^{1}H COSY (C) and ^{1}H-^{13}C HMBC (D) of Fr-1. I, Fuc_{2S4S}; II, Fuc_{3S4S}; U, GlcA; U_{I} & U_{II}, U substituted with I or II, respectively; T, anTal-diol; T_{I} & T_{II}, T linked with U_{I} or U_{II}, respectively.

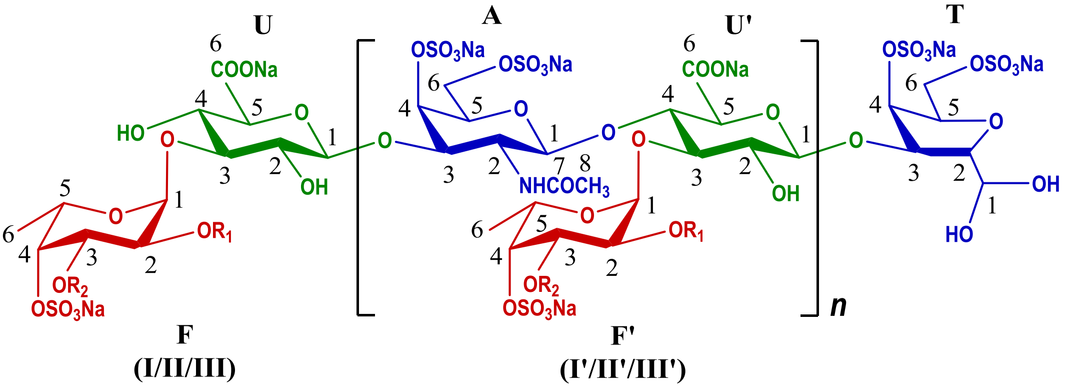

R_1= -SO_3Na, R_2= -H in I & I'; R_1= -H, R_2= -SO_3Na in II & II'; R_1= R_2= -H in III & III';

Fr-2**,** n = 1; Fr-3**,** n = 2; Fr-4**,** n = 3.

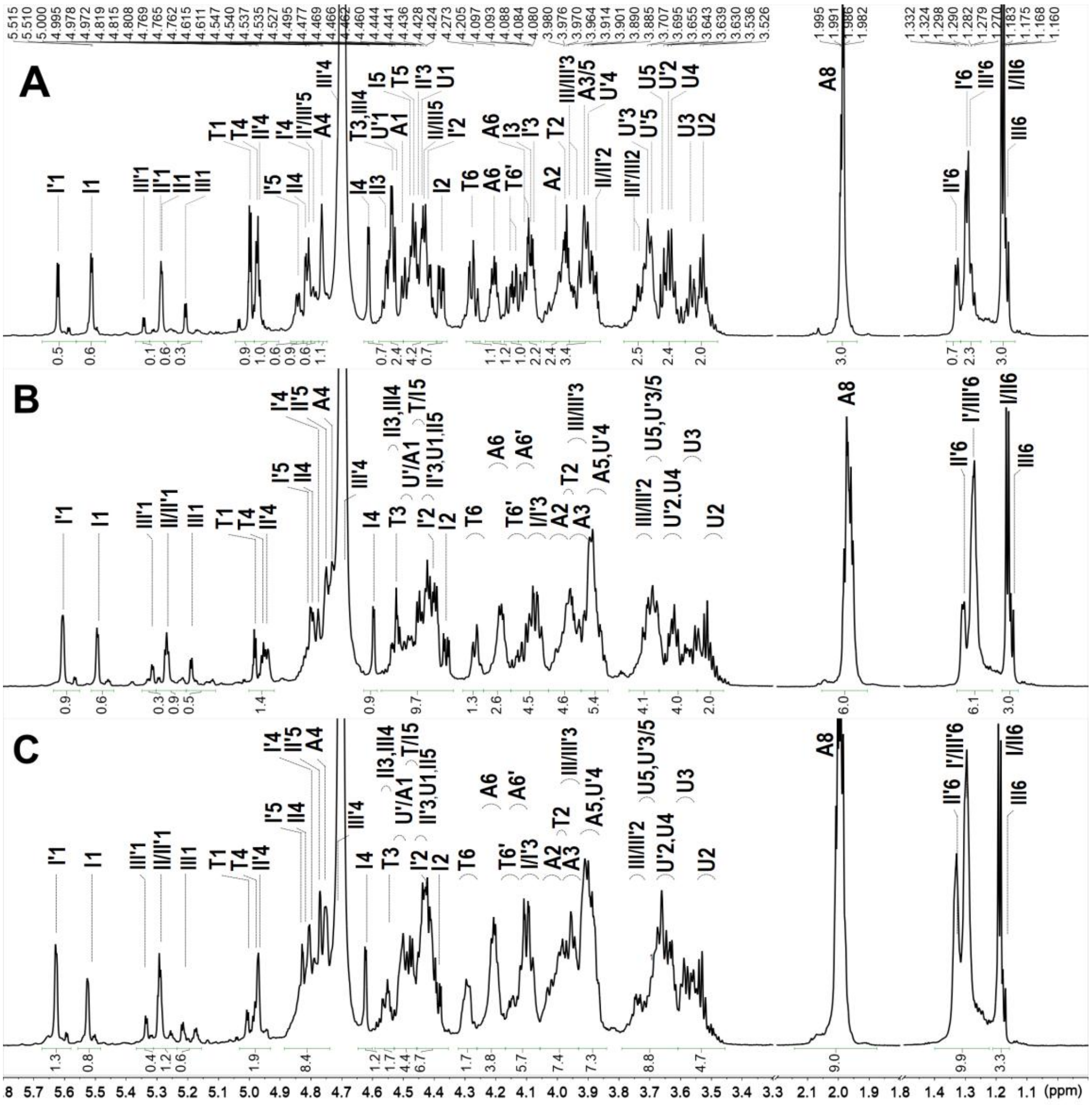

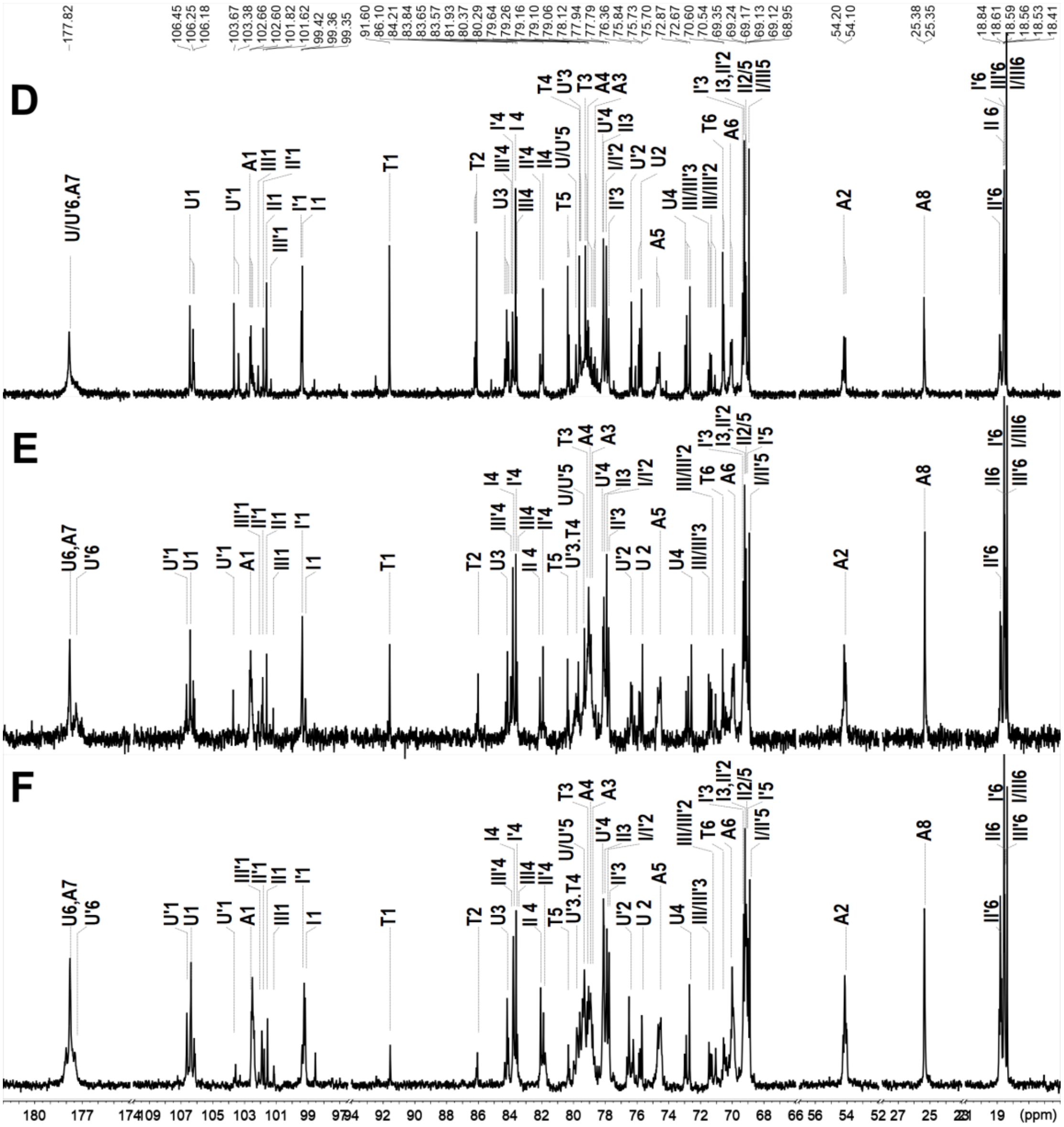

Figure S4. Structure formula, ^{1}H NMR (A,B,C), ^{13}C NMR (D,E,F) spectra of Fr-2 (A, D)**,** Fr-3 (B, E) and Fr-4 (C, F). Marked symbols are the same as shown in structure formula.

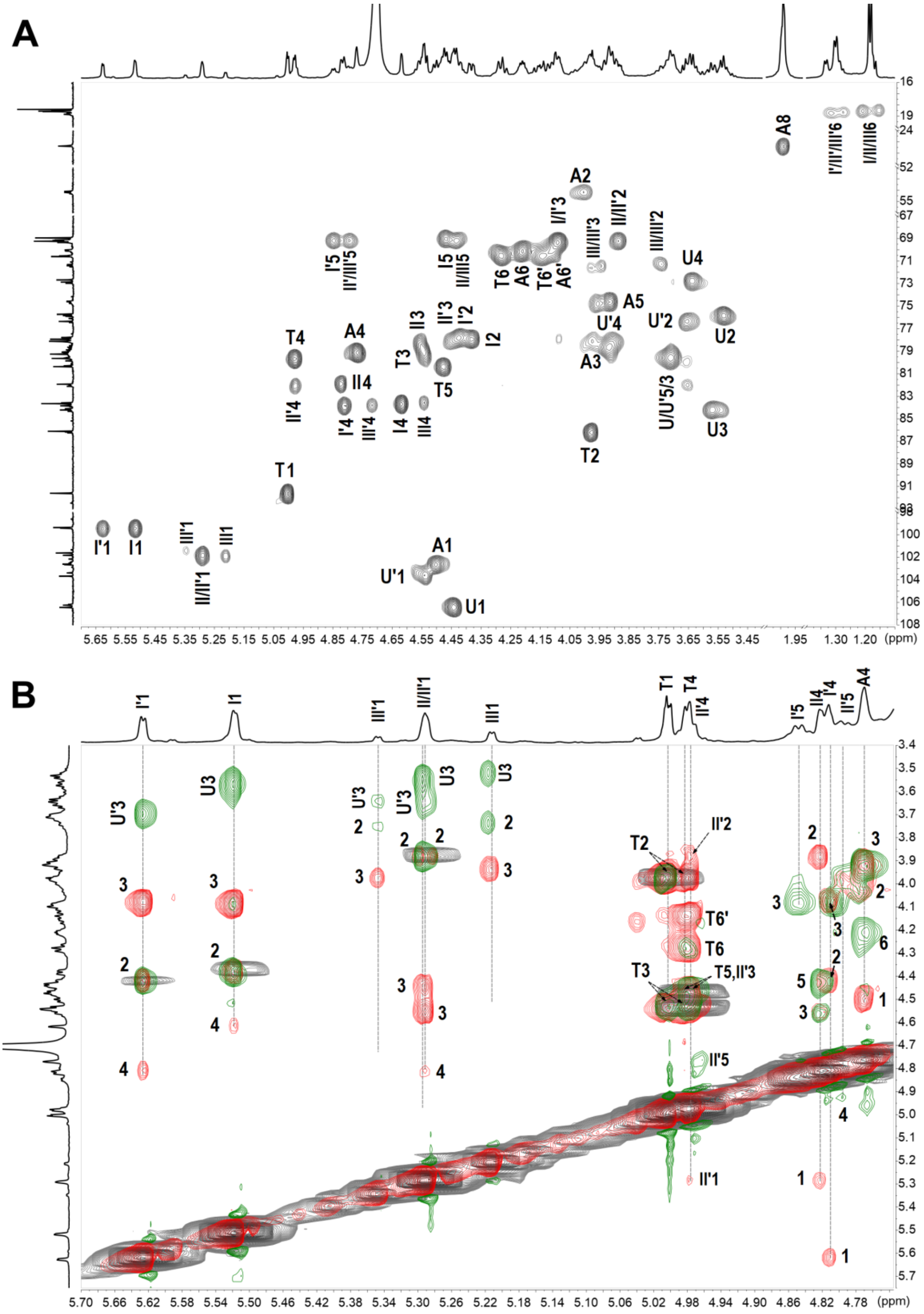
A
B
(ppm)
A8
A2
A1
A3
A4
A5
A6
A6'
T1
T2
T3
T4
T5
T6
T6'
U1
U2
U3
U4
U'1
U'2
U'4
U/U'5/3
I1
I'1
I2
I'2
I3
I'3
I4
I'4
I5
I'5
II/II'1
III'1
III1
II3
II'3
II4
II'4
III4
III'4
I/I'3
II/II'2
III/III'2
III/III'3
II/III5
II'/III'5
I'/II'/III'6
I/II/III6
I/II'1
T5,II'3
II'2
II'5
II'1

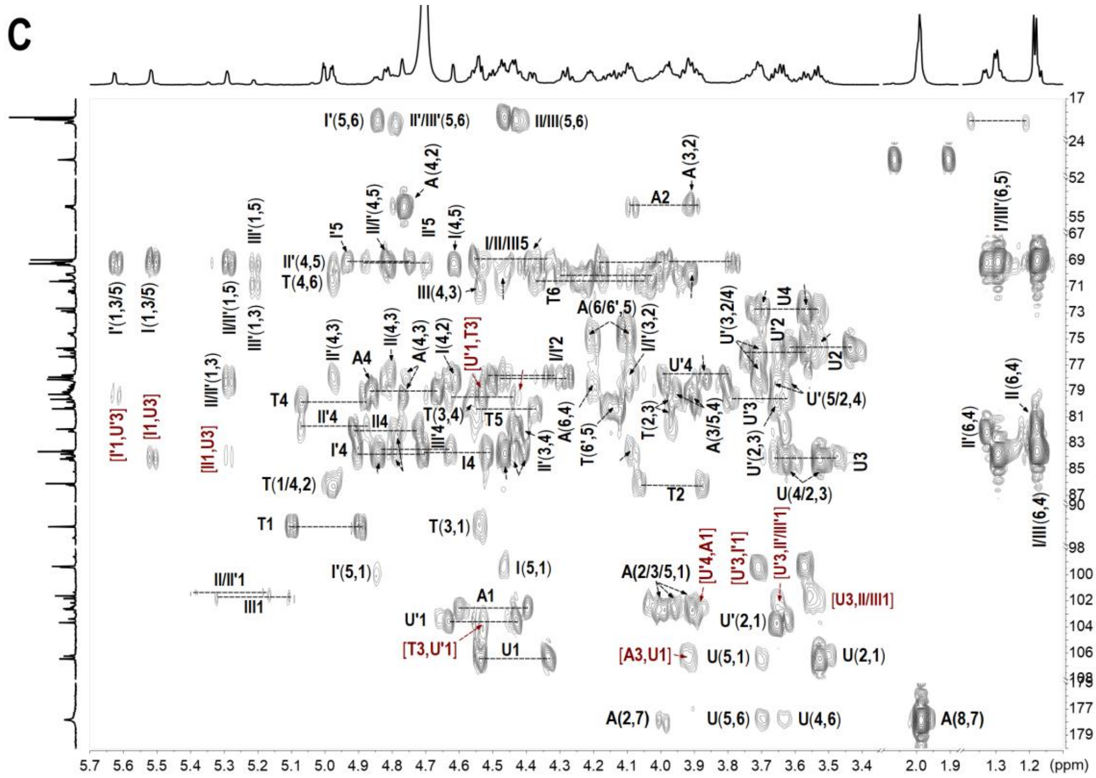

Figure S5. ^{1}H-^{13}C HSQC (A), superposition of ^{1}H-^{1}H COSY (black), TOCSY (red), and ROESY (green) (B), ^{1}H-^{13}C HMBC (C) spectra of Fr-2. Marked symbols are the same as in Figure S3.

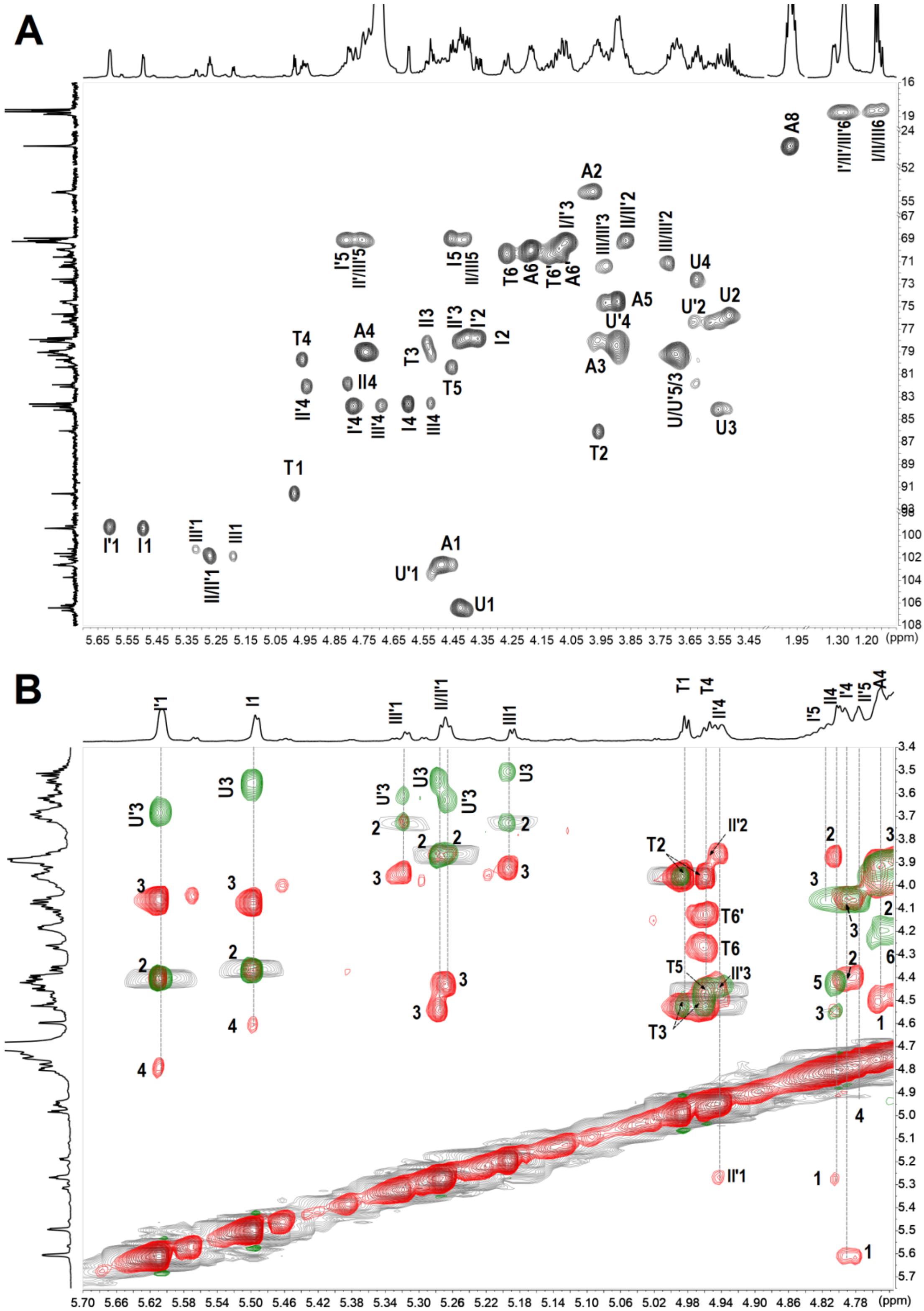

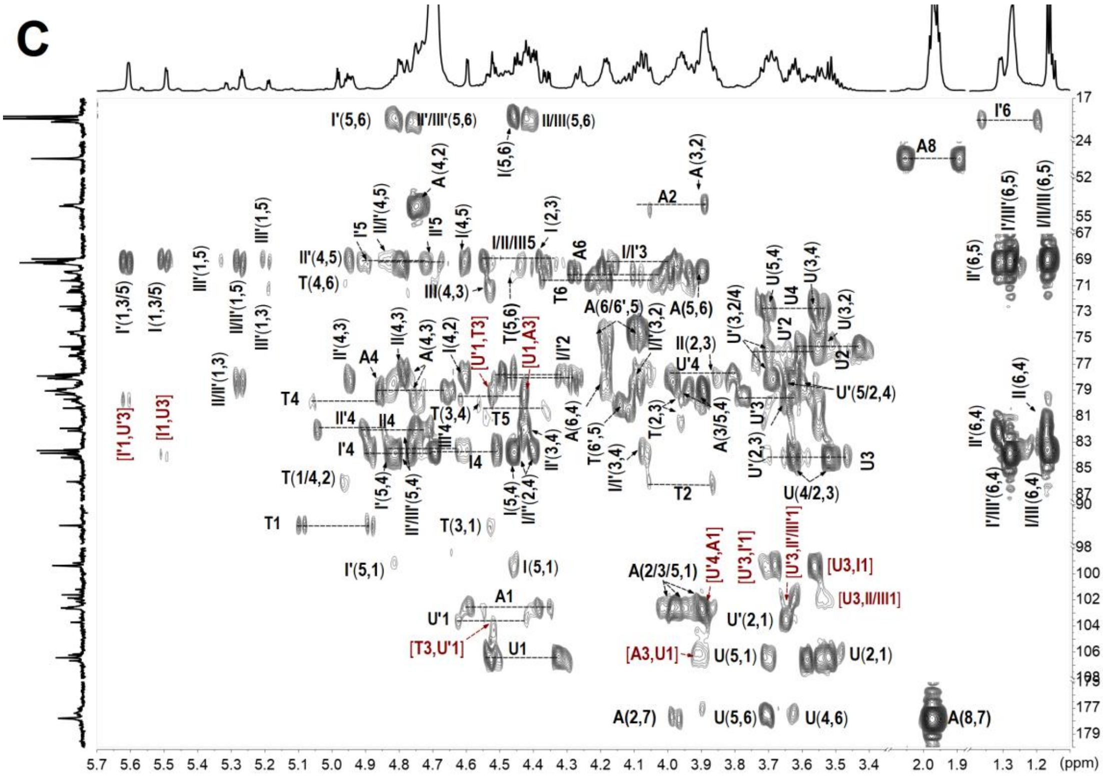

Figure S6. ^{1}H-^{13}C HSQC (A), superposition of ^{1}H-^{1}H COSY (black), TOCSY (red), and ROESY (green) (B), ^{1}H-^{13}C HMBC (C) spectra of Fr-3. Marked symbols are the same as in Figure S3.

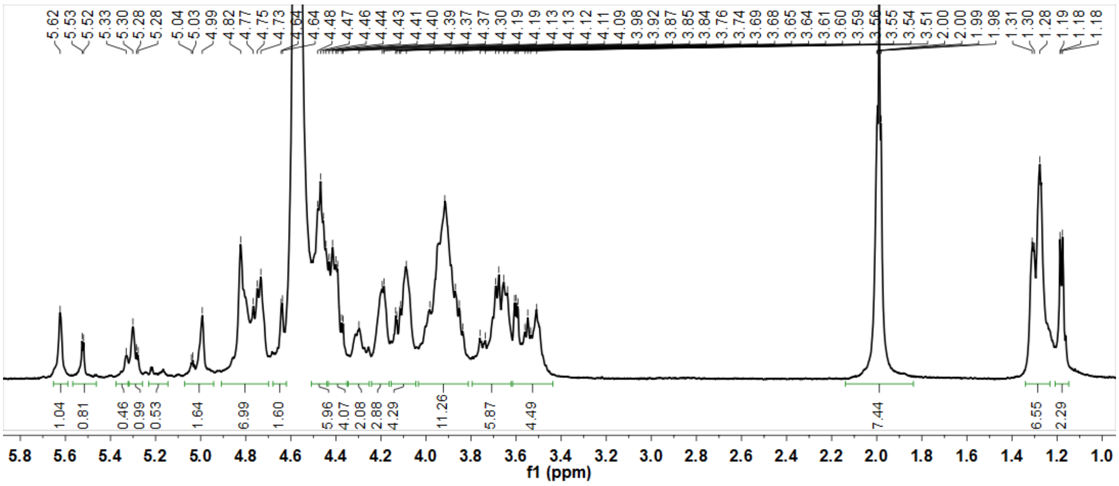

Figure S7. ^{1}H-NMR spectrum of fraction 5.

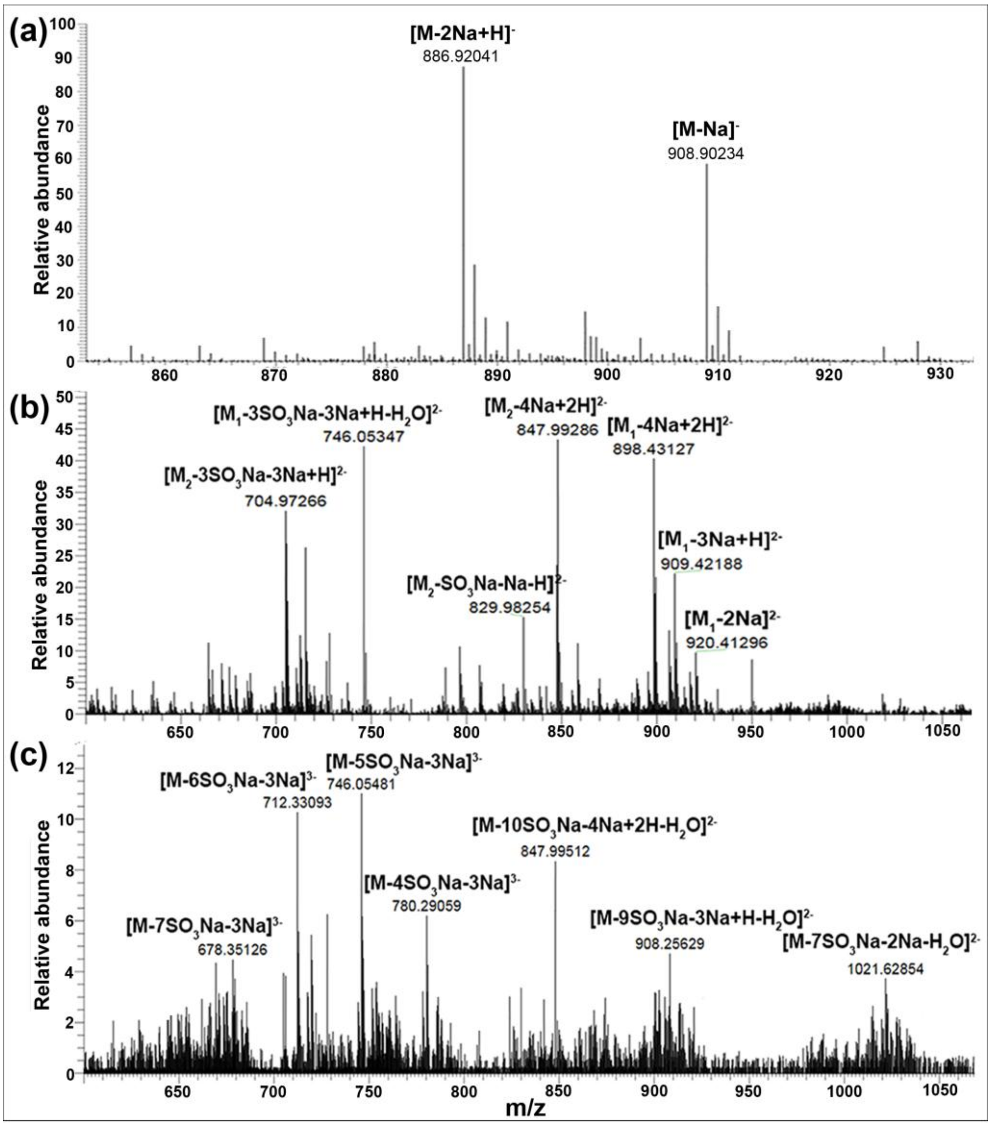

Figure S8. ESI-Q-TOF MS spectra of Fr-1 (A), Fr-2 (B), and Fr-3 (C).

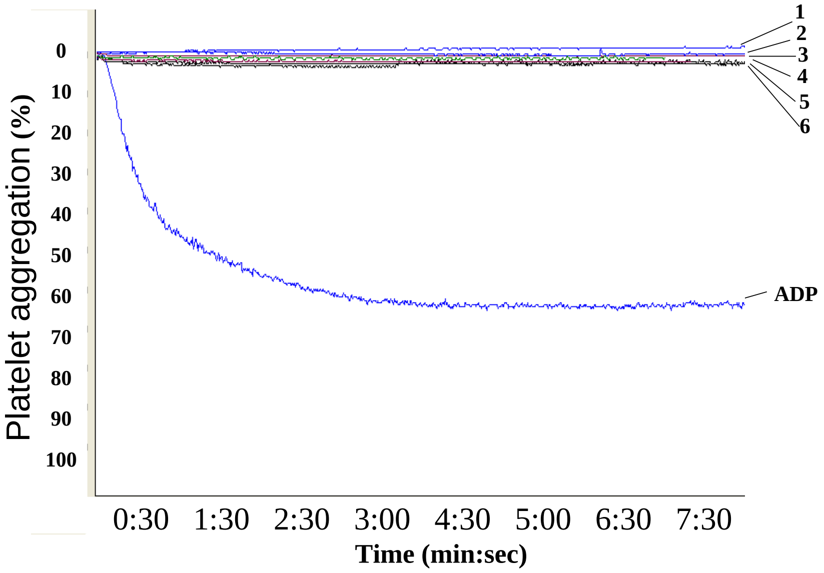

Figure S9. Effects of Fr-1–Fr-4 (1–4), AjFG (5), and dAjFG (6) (50 μg/mL) on platelet aggregation (n = 2).

Table S1. $^{1}H/^{13}C$ NMR chemical shift assignments of Fr-2. (δ, ppm; J, Hz)

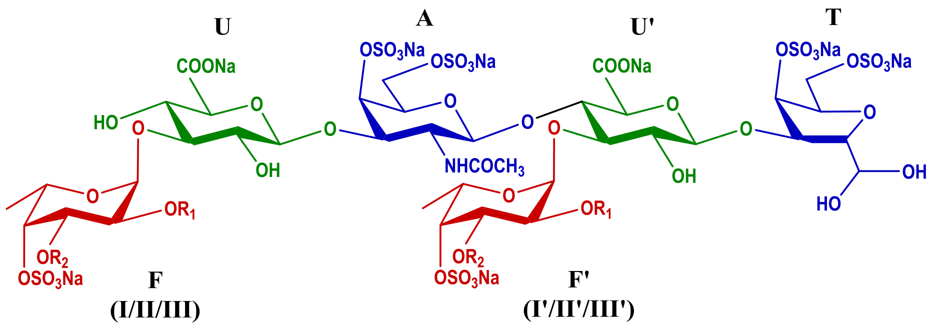

R_1= -SO_3Na, R_2= -H in I & I'; R_1= -H, R_2= -SO_3Na in II & II'; R_1= R_2= -H in III & III'.

		δ	**Couplings**		δ			δ	**Couplings**		δ
**I**	H-1	5.512	$J_{(1,2)}$ = 4.00	C-1	99.36	**U**	H-1	4.442	$J_{(1,2)}$ = 8.64	C-1	106.45<br>106.25
	H-2	**4.377**	$J_{(2,3)}$ = 10.56	C-2	**77.94**		H-2	3.526	$J_{(2,3)}$ = 8.96	C-2	75.72
	H-3	4.088	$J_{(3,4)}$ = 3.20	C-3	69.24		H-3	3.569	$J_{(3,4)}$ = 9.20	C-3	84.21
	H-4	**4.613**	--	C-4	83.65		H-4	3.629	$J_{(4,5)}$ = 8.32	C-4	72.66<br>72.89
	H-5	4.470	$J_{(5,6)}$ = 6.56	C-5	68.95		H-5	3.660		C-5	79.85
	H-6	1.178		C-6	18.41					C-6	177.82
**II**	H-1	5.285	$J_{(1,2)}$ = 4.16	C-1	101.62	**A**	H-1	4.500	$J_{(1,2)}$ = 8.24	C-1	102.66<br>102.60
	H-2	3.878	--	C-2	69.17		H-2	4.003	--	C-2	54.20
	H-3	**4.553**	--	C-3	**78.12**		H-3	3.974<br>3.918	--	C-3	78.83<br>78.64
	H-4	**4.817**	--	C-4	81.93		H-4	**4.765**	--	C-4	**79.03**
	H-5	4.433	$J_{(5,6)}$ = 6.56	C-5	69.12		H-5	3.956<br>3.915		C-5	74.75<br>74.56
	H-6	1.178		C-6	18.60		H-6	**4.205**	$J_{(5,6)}$ = 3.20	C-6	70.06
							H-6'	**4.105**<br>**4.095**	$J_{(6,6')}$ =11.52	C-7	177.82
							H-8	1.986<br>1.982		C-8	25.38<br>25.35
**III**	H-1	5.207	$J_{(1,2)}$ = 4.08	C-1	102.15						
	H-2	3.742	--	C-2	71.32						
	H-3	3.945	--	C-3	71.50						
	H-4	**4.540**	--	C-4	**83.57**						
	H-5	4.433	$J_{(5,6)}$ = 6.62	C-5	68.95						
	H-6	1.163		C-6	18.41						
**I'**	H-1	5.621	$J_{(1,2)}$ = 4.00	C-1	99.42	**U'**	H-1	4.533	$J_{(1,2)}$ = 7.52	C-1	103.67<br>103.38
	H-2	**4.420**	--	C-2	**77.94**		H-2	3.639	--	C-2	76.36
	H-3	4.082	$J_{(3,4)}$ = 2.80	C-3	69.85		H-3	3.707	--	C-3	79.61
	H-4	**4.807**		C-4	**83.85**		H-4	3.918	--	C-4	**78.14**
	H-5	4.843	$J_{(5,6)}$ = 6.40	C-5	69.17		H-5	3.693		C-5	79.85

	H-6	1.295		C-6	18.56						C-6	177.82
**II’**	H-1	5.286	--	C-1	101.82		**T**	H-1	**4.998**	$J_{(1,2)}$ = 4.26	C-1	91.61
	H-2	3.880	--	C-2	69.24			H-2	3.978	--	C-2	86.10
	H-3	**4.451**	--	C-3	**77.78**			H-3	4.536	--	C-3	79.26
	H-4	**4.967**	--	C-4	**82.11**			H-4	**4.975**	$J_{(4,5)}$ = 4.88	C-4	79.64
	H-5	4.794	$J_{(5,6)}$ = 6.40	C-5	69.12			H-5	4.476	--	C-5	80.37
	H-6	1.328		C-6	18.85			H-6	4.277<br>4.265	$J_{(5,6)}$ = 2.32	C-6	70.60<br>70.53
								H-6’	4.153<br>4.134	$J_{(6,6')}$ =11.44		
**III’**	H-1	**5.342**	$J_{(1,2)}$ = 4.16	C-1	101.33							
	H-2	3.742	--	C-2	71.08							
	H-3	3.976	--	C-3	71.40							
	H-4	**4.718**		C-4	**83.93**							
	H-5	4.784	$J_{(5,6)}$ = 6.48	C-5	69.12							
	H-6	1.275		C-6	18.53							

Table S2. $^1H/^{13}C$ NMR chemical shift assignments of Fr-3. (δ, ppm; J, Hz)

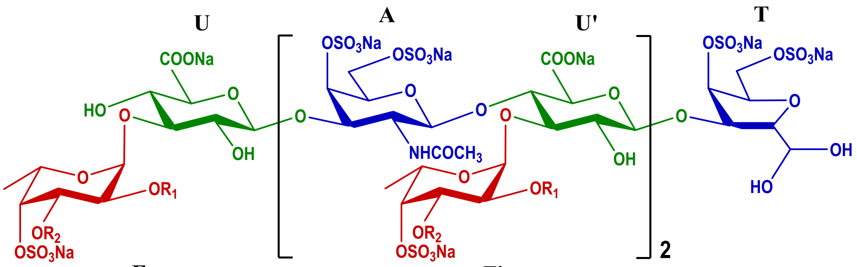

F
(I/II/III)

F'
(I'/II'/III')

R_1= -SO_3Na, R_2= -H in I & I'; R_1= -H, R_2= -SO_3Na in II & II'; R_1= R_2= -H in III & III'.

		δ		*δ*				*δ*		*δ*
**I**	H-1	5.492	C-1	99.17		**U**	H-1	4.426	C-1	106.43<br>106.23
	H-2	**4.361**	C-2	**77.91**			H-2	3.518	C-2	75.66
	H-3	4.073	C-3	69.22			H-3	3.555	C-3	84.17
	H-4	**4.596**	C-4	83.83			H-4	3.627	C-4	72.57<br>72.77
	H-5	4.456	C-5	68.92			H-5	3.630	C-5	79.32
	H-6	1.163	C-6	18.38					C-6	177.79
**II**	H-1	5.271	C-1	101.61		**A**	H-1	4.491	C-1	102.62<br>102.54
	H-2	3.866	C-2	69.13			H-2	3.978	C-2	54.18<br>54.07
	H-3	**4.535**	C-3	**78.09**			H-3	3.963	C-3	78.90
	H-4	**4.796**	C-4	82.12			H-4	**4.731**	C-4	**79.05**
	H-5	4.413	C-5	69.13			H-5	3.933<br>3.895	C-5	74.69<br>74.53
	H-6	1.163	C-6	18.53			H-6	**4.181**	C-6	69.88
							H-6'	**4.102**	C-7	177.79
							H-8	1.973	C-8	25.34<br>25.32
**III**	H-1	5.188	C-1	101.19						
	H-2	3.723	C-2	71.29						
	H-3	3.934	C-3	71.49						
	H-4	**4.525**	C-4	**83.56**						
	H-5	4.413	C-5	69.00						
	H-6	1.148	C-6	18.38						
**I'**	H-1	5.605	C-1	99.36		**U'**	H-1	4.518	C-1	103.72<br>106.66
	H-2	**4.403**	C-2	**77.91**			H-2	3.639<br>3.581	C-2	76.39<br>76.31
	H-3	4.073	C-3	69.30			H-3	3.699	C-3	79.85
	H-4	**4.776**	C-4	**83.63**			H-4	3.899	C-4	**78.14**

	H-5	4.817	C-5	69.57			H-5	3.685	C-5	79.32
	H-6	1.275	C-6	18.56					C-6	177.40
**II'**	H-1	5.265	C-1	101.86		**T**	H-1	**4.980**	C-1	91.59
	H-2	3.866	C-2	69.22			H-2	3.959	C-2	86.02
	H-3	**4.406**	C-3	**77.78**			H-3	4.523	C-3	79.13
	H-4	**4.943**	C-4	**81.92**			H-4	**4.956**	C-4	79.69
	H-5	4.751	C-5	68.92			H-5	4.455	C-5	80.38
	H-6	1.308	C-6	18.80			H-6	4.266	C-6	70.60
							H-6'	4.130		
**III'**	H-1	**5.314**	C-1	102.13						
	H-2	3.723	C-2	71.05						
	H-3	3.934	C-3	71.37						
	H-4	**4.690**	C-4	**83.96**						
	H-5	4.762	C-5	69.00						
	H-6	1.267	C-6	18.50						
